# Three‐Dimensional Transjugular Intrahepatic Portosystemic Shunt Geometry Predicts Shunt Dysfunction

**DOI:** 10.1111/apt.70133

**Published:** 2025-04-09

**Authors:** Carsten Meyer, Markus Kimmann, Katharina Böhm, Sebastian Nowak, Alba Maria Paar Pérez, Jörn Arne Meier, Sara Noemi Reinartz Groba, Juliana Gödiker, Frank Erhard Uschner, Feras Sanoubara, Johannes Chang, Jonel Trebicka, Alois Martin Sprinkart, Michael Praktiknjo

**Affiliations:** ^1^ Department of Diagnostic and Interventional Radiology University of Bonn Bonn Germany; ^2^ Department of Internal Medicine B University of Münster Münster Germany; ^3^ Department of Internal Medicine I University of Bonn Bonn Germany; ^4^ European Foundation for the Study of Chronic Liver Failure—EF CLIF Barcelona Spain

**Keywords:** cirrhosis, portal hypertension, transjugular intrahepatic portosystemic shunt

## Abstract

**Background:**

Patients with decompensated cirrhosis are at risk of portal hypertension‐related complications, such as refractory ascites or variceal bleeding. Transjugular intrahepatic portosystemic shunt (TIPS) insertion is the most effective treatment to reduce portal hypertension. However, patients are at risk for TIPS dysfunction.

**Aims:**

We aimed to investigate the prognostic value of three‐dimensional (3D) TIPS geometry in predicting TIPS dysfunction.

**Methods:**

A total of 107 patients who underwent TIPS insertion between 2014 and 2019 and received a computed tomography (CT) scan after TIPS insertion during routine clinical practice were included. We used a semiautomated algorithm and multiplanar reconstructions of these CT scans to calculate parameters of 3D TIPS geometry. The primary outcome of this study was the development of TIPS dysfunction (defined as need for invasive TIPS revision). To identify predictors for the development of TIPS dysfunction, Cox regression analyses were performed with TIPS dysfunction as the endpoint.

**Results:**

Thirty‐two patients developed TIPS dysfunction and were compared to the dysfunction‐free 75 patients. A larger distance from the cranial TIPS stent end to the vena cava inferior (*p* < 0.001, HR 1.061, 95% CI 1.030–1.093) and the maximum stent curvature (*p* = 0.003, HR 1.020, 95% CI 1.007–1.034) were significantly associated with TIPS dysfunction in a multivariate Cox regression analysis.

**Conclusion:**

A more pronounced stent curvature and a longer cranial stent distance from the inferior vena cava were identified as independent predictors of TIPS dysfunction. Interventionalists should choose a more central and less curved TIPS tract during the TIPS procedure to reduce the risk of development of TIPS dysfunction.

**Trial Registration:**

This retrospective monocentric study includes patients from the NEPTUN cohort (registered at ClinicalTrials.gov; Identifier: NCT03628807).

## Graphical abstract

Regarding the role of 3D TIPS geometry in predicting TIPS dysfunction, this study identified a larger distance of the cranial stent end from the inferior vena cava and a larger stent curvature as predictors of dysfunction. Therefore, a less curved, more central TIPS is recommended to reduce the risk of dysfunction

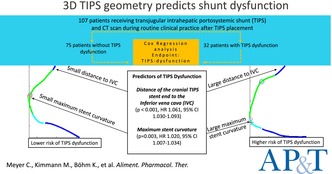

**Abbreviations**
2D: (two dimensional)
3D: (three dimensional)
95% CI: (95% confidence interval)
CT: (computed tomography)
CV: (central venous)
HE: (hepatic encephalopathy)
HR: (hazard ratio)
INR: (international normalised ratio)
IVC: (inferior vena cava)
MELD: (model of end‐stage liver disease)
PSPG: (portosystemic pressure gradient)
PTFE: (polytetrafluorethylene)
PV: (portal venous)
TIPS: (transjugular intrahepatic portosystemic shunt)
VCX: (VIATORR controlled expansion)
VTS: (VIATORR TIPS)

## 1 Introduction

Liver cirrhosis with its severe complications caused by portal hypertension, such as variceal bleeding and refractory ascites, is a major healthcare burden. Patients with liver cirrhosis suffer from high rates of hospitalisation as well as increased morbidity and mortality [1].

Variceal bleeding and refractory ascites, as severe complications of portal hypertension, can be treated by implantation of a transjugular intrahepatic portosystemic shunt (TIPS). TIPS reduces the portosystemic pressure gradient by partially redirecting the portal venous blood flow to the inferior vena cava (IVC) [2]. In selected patients, TIPS can improve the outcome of patients with decompensated cirrhosis [3, 4, 5, 6, 7].

In the early era of bare metal stents, TIPS dysfunction was a major complication in up to 80% in 2 years [8, 9]. Technical advancements led to resolving several technical problems. However, the introduction of polytetrafluoroethylene (PTFE)‐covered stents in the millennials marked an important milestone, which reduced but did not abolish the problem of TIPS stent dysfunction [2, 10, 11, 12], which can have devastating clinical impacts such as variceal rebleeding or recurrent ascites.

TIPS dysfunction, including stenosis and occlusion, among other factors [13, 14, 15, 16], seems to be influenced by the haemodynamic flow characteristics [17]. These are discussed to be influenced by the geometry and position of the TIPS stent. In the modern era of PTFE‐covered TIPS stents, some studies suggested that characteristics of TIPS stent geometry and position, such as portal venous inflow angles and distance from the cranial stent end to the IVC, might predict TIPS dysfunction [18]. However, the data on especially two‐dimensional (2D) assessed parameters are conflicting and do not seem to sufficiently reflect the complex portal venous anatomy [19, 20]. Data on three‐dimensional (3D) assessed parameters are scarce because CT scan is not routinely performed after the TIPS procedure. Moreover, manual 3D reconstruction and the measurements of geometrical parameters are time‐consuming procedures for healthcare professionals.

This study aimed to develop a semiautomated algorithm to assess 3D parameters and characteristics of TIPS geometry and to evaluate their predictive value for the development of TIPS dysfunction.

## 2 Materials and Methods

### 2.1 Study Population

**FIGURE 1:**
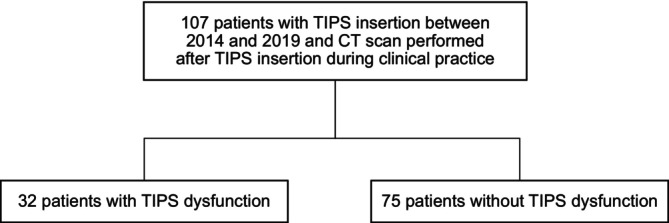
Flow chart of study population.

This retrospective monocentric study includes patients from the NEPTUN cohort (registered at ClinicalTrials.gov; Identifier: NCT03628807), who underwent the TIPS procedure between 2014 and 2019 and received a CT scan after TIPS insertion during routine clinical practice. Follow‐up included evaluation of TIPS function using noninvasive imaging such as CT and ultrasound as well as clinical condition and standard biochemical blood analysis. Inclusion criteria were patient age of 18 or more and first‐time implantation of a PTFE‐covered TIPS stent for refractory ascites or variceal bleeding. Overall, 107 patients were eligible for this study (Figure 1). The primary endpoint was the development of TIPS dysfunction. TIPS dysfunction was suspected in the case of clinical signs like recurrence of ascites or variceal bleeding and followed by noninvasive tests to objectify TIPS dysfunction such as Doppler ultrasound with proof of reduction or nondetectability of flow in the stent and/or CT scan with signs of stent occlusion. Overall, the criteria used to define stent dysfunction were the same for all patients and based on the need for invasive revision of the TIPS. In the case of invasive visualisation with angiography without the need for invasive revision of the TIPS, we did not document the endpoint of TIPS dysfunction. Secondary endpoints were development of overt hepatic encephalopathy (HE) after TIPS in the entire cohort as well as detection of ascites at least 30 days after TIPS insertion in the subgroup of patients with refractory ascites as TIPS indication.

The ethical review board of the University Hospital of Bonn approved the study (positive ethical review board vote 038/20).

Written, informed consent was obtained from each patient included in the study.

The study protocol conforms to the ethical guidelines of the 1975 Declaration of Helsinki as reflected in a priori approval by the institution's human research committee.

### 2.2 TIPS Procedure

An experienced team of hepatologists and radiologists performed the TIPS procedure to reduce the portosystemic pressure gradient (PSPG) under ultrasound and fluoroscopic guidance as previously described [21, 22, 23]. In summary, a tract was formed from the hepatic vein to the portal vein. The PSPG was measured before and after implantation of a PTFE‐covered stent (W.L. Gore Medical). The types of stent grafts used in this study were the VIATORR TIPS (VTS) and the VIATORR controlled expansion (VCX) stent grafts.

The diameter of the stent was set at the interventionist's discretion according to PSPG measurement.

### 2.3 Assessment of TIPS Geometry

First, multiplanar CT reconstructions were created using a proprietary programme (Intellispace; Philips Healthcare GmbH, Hamburg, Germany). Based on these MPRs, the evaluation was carried out by applying a dedicated software written in MATLAB (Mathworks). A detailed explanation of the algorithm can be found in the Supporting Information 1.

**FIGURE 2:**
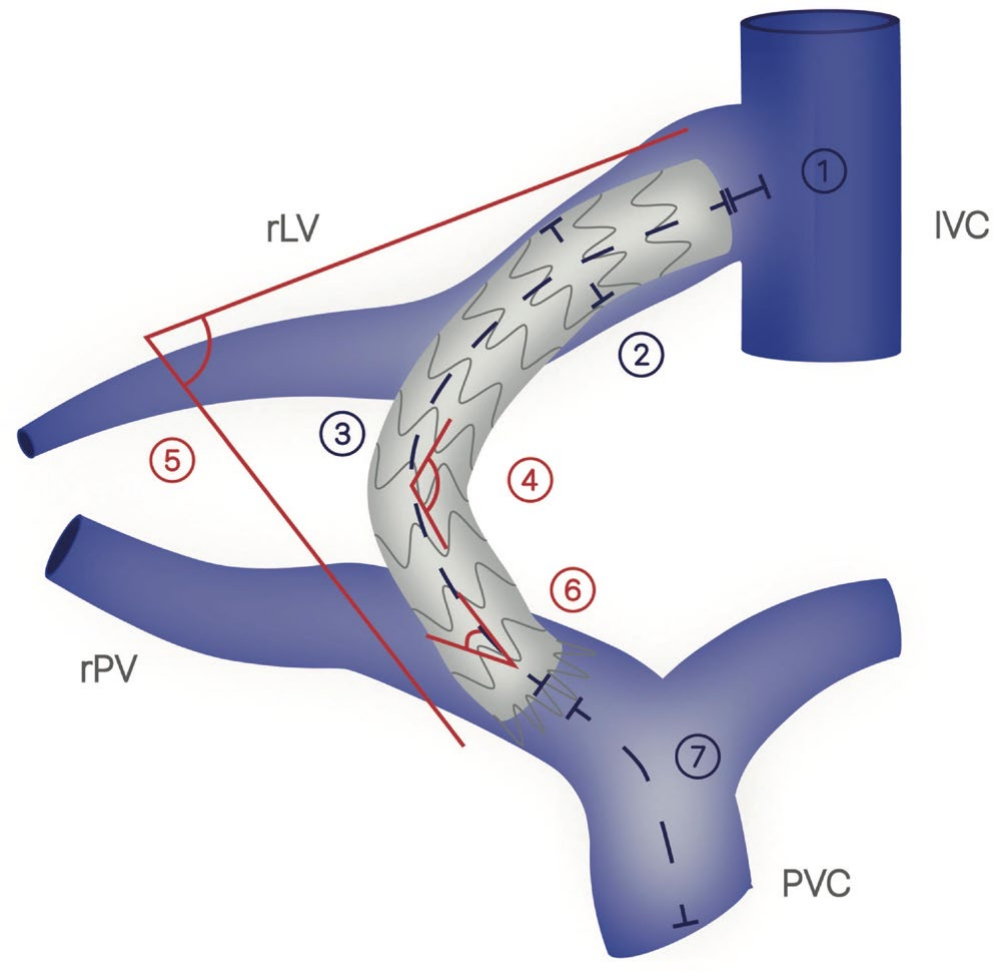
Schematic illustration of the 3D TIPS geometry parameters. 3D cranial TIPS stent end (mm): Length from the cranial TIPS stent end in the liver vein to the IVC; 3D minimal stent diameter (mm): Smallest diameter in the entire TIPS stent; 3D length of covered stent portion (mm): Length from the beginning of the distal covered stent end to the end of the cranial covered stent end; 3D stent curvature (degrees/cm): Result of segmentation of the path within the TIPS stent into straight lines with gaps of 5 mm and angle measurements over 1 cm. The parameter describes the maximum change in direction between these sections within the entire stent; 3D angle between the covered stent ends (degrees): Angle between two straight lines orthogonal to the covered stent ends; 3D α angle (degrees): Angle formed between the beginning of the covered stent in the PV to the course of the PV; 3D confluence to TIPS stent (mm): Length from the beginning of the path to the beginning of the uncovered distal stent end.

In short, a venous path was generated starting from the beginning of the portal venous confluence through the TIPS to the IVC using multiplanar reconstructions orthogonal to the path, and different parameters of geometry were obtained (Figure 2).

- 3D cranial TIPS stent end (mm): distance of the cranial stent end to the IVC.
- 3D minimal stent diameter (mm): smallest diameter of the TIPS stent.
- 3D length of covered stent portion (mm): length from the beginning of the distal covered stent end to the end of the cranial covered stent end.
- 3D stent curvature (degrees/cm): Result of segmentation of the path within the TIPS stent into straight lines with gaps of 5 mm and angle measurements over 1 cm. The parameter describes the maximum change in direction between these sections within the entire stent. If the stent is straight, the value would be 0. Example: If the path makes a bend of 90° within one of the stent sections, the value would be 90.
- 3D angle between the covered stent ends (degrees): angle between two straight lines orthogonal to the covered stent ends.
- 3D α angle (degrees) angle between the caudal beginning of the covered stent in the PV and the course of the PV.
- 3D confluence to TIPS (mm): length from the beginning of the path to the beginning of the uncovered distal stent end.

### 2.4 Statistical Analysis

SPSS (version 24, IBM, Armonk, NY, USA) was used to analyse all data. Descriptive statistics were performed for all variables. Categorical variables are presented in absolute cases and per cent, while continuous variables are presented as median with interquartile range. Nonparametric testing (Qui‐square test for categorical variables, Mann–Whitney U test for continuous variables) was used to compare the groups with and without TIPS dysfunction. Uni‐ and multivariate Cox regression analysis with TIPS dysfunction as the endpoint was performed to identify predictors of TIPS dysfunction. To evaluate the relevance of the time from TIPS insertion to CT acquisition, an additional Cox regression analysis was performed, including the parameters that were significantly associated with TIPS dysfunction in the multivariate Cox regression analysis, as well as the time from TIPS insertion to CT acquisition. Furthermore, we performed an additional Cox regression analysis with overt HE as the endpoint, including the parameters that were significantly associated with TIPS dysfunction in the previous analysis. Additionally, a Cox regression analysis was performed in the subgroup of patients with refractory ascites as the indication for TIPS, with detection of ascites 30 days after TIPS insertion as the endpoint. All parameters with *p*‐values < 0.1 in the univariate analysis were considered potential risk factors and therefore included in the multivariate model. The multivariate Cox regression analysis was performed using forward stepwise logistic regression, excluding all parameters with *p*‐values > 0.1. In case of missing parameters within the Cox regression model, the case was excluded for this particular analysis. *p*‐Values below 0.05 were considered statistically significant.

## 3 Results

### 3.1 General Patient Characteristics at Baseline

This study included 107 patients; 57 (53.3%) patients were male. The median age was 58 (51–65). Most patients had alcohol‐related liver cirrhosis (*n* = 63; 58.9%). The main indications for TIPS were refractory ascites (*n* = 60; 56.15%) and variceal bleeding (*n* = 37, 34.6%). Eighty patients (74,8%) presented with ascites at the time of TIPS placement. In the entire cohort, 61 (57%) of patients received the VTS stent graft and 46 (43%) of patients received the VCX stent graft.

Overall, 32 patients developed TIPS dysfunction. The median times were 539 (127.75–985) days from TIPS procedure to TIPS dysfunction, 337 (29–612) days from TIPS procedure to CT acquisition and 134 (4.75–505.5) days from CT acquisition to TIPS dysfunction. The TIPS dysfunction group had a median age of 58 (51–64) and included 15 (46.9%) men. The main indication for TIPS in the TIPS dysfunction group was refractory ascites (*n* = 21; 65.6%), and the main aetiology of cirrhosis was alcohol related (*n* = 19; 59.38%). In the dysfunction group, 20 (62.5%) received a VTS stent graft, while 12 (37.5%) received a VCX stent graft. The group without TIPS dysfunction had a median age of 58 (51–65) and included 42 (*n* = 56%) male patients; 41 (54.7%) of patients received a VTS stent graft and 34 (45.3%) received a VCX stent graft. Both groups were comparable in terms of general characteristics, medical history, scores, hepatic haemodynamics before and after TIPS and distribution of used stent grafts (VCX and VTS) (Table 1 and Table 2). Noteworthy, the median portosystemic pressure gradient (PSPG) before TIPS revision in the TIPS dysfunction group was 18 mmHg [13, 14, 15, 16, 17, 18, 19, 20, 21, 22, 23, 24, 25].

**TABLE 1:** Study population characteristics at baseline.

Parameter		All (*n* = 107)	No dysfunction (*n* = 75)	TIPS dysfunction (*n* = 32)	*p*
General characteristics	Age (years)	58 (51–65)	58 (51–65)	58 (51–64)	0.911
	Sex (male/female)	57/50 (53.3%/46.7%)	42/33 (56%/44%)	15/17 (46.9%/53.1%)	0.386
	TIPS indication (variceal bleeding/refractory ascites/other)	37/60/10 (34.6%/56.15/9.3%)	27/29/9 (36%/52%/12%)	10/21/1 (31.3%/65.6%/3.1)	0.249
	Aetiology of cirrhosis (alcohol related/other)	63/44 (58.9%/41.1%)	44/31 (58.67%/41.33%)	19/13 (59.38%/40.62%)	0.946
Medical history	Ascites (yes)	81 (76.41%)	57 (77.03%)	24 (75%)	0.821
	Variceal bleeding (yes)	45 (42.45%)	33 (44.59%)	12 (37.5%)	0.498
	Overt hepatic encephalopathy (yes)	17 (16.19%)	14 (18.67%)	3 (10.0%)	0.276
	Oesophageal varices grade (no/1/2/3/4)	9/29/38/18/2 (9.38%/30.2%/39.58%/18.75%/2.08%)	6/19/28/14/1 (8.82%/27.94%/41.18%/20.59%/ 1.47%)	3/10/10/4/1 (10.71%/35.71%/35.71%/14.29%/3.57%)	0.833
Scores	MELD	10 (8–13)	10 (8–14)	10.5 (8–12)	0.509
	MELD‐Na	21 (20–23)	21 (20–24)	21.5 (20–23)	0.579
	Child–Pugh score	9 (7–10)	9 (8–10)	8 (7–9.75)	0.257
	Child–Pugh grade (A/B/C)	14/61/32 (13.1%/57%/29.9%)	8/43/24 (10.7%/57.3%/32%)	6/18/8 (18.8%/56.3%/25%)	0.474
Hepatic haemodynamics	CV‐pressure before TIPS	6 (4–10)	7 (3–10.5)	6 (4–9.25)	0.705
	PV‐pressure before TIPS	26.5 (23–32)	26 (23–31.25)	27 (24–32.25)	0.548
	PSPG before TIPS	20 (16–23)	20 (16–23.5)	20 (18–23)	0.779
	CV‐pressure after TIPS	9 (7–13)	9 (7–12)	11 (7–15.25)	0.250
	PV‐pressure after TIPS	19 (15–22)	19 (14.75–22)	20 (17–24.25)	0.294
	PSPG after TIPS	8 (6–10.75)	8 (6.5–10.5)	7 (5–11)	0.599

*Note:* Categorical variables are presented in absolute cases and per cent while continuous variables are presented as median with interquartile range. Nonparametric testing (Qui‐square test for categorical variables and Mann–Whitney U test for continuous variables).

Abbreviations: CV‐pressure, central venous pressure (mmHg); MELD, model for end‐stage liver disease; PSPG, portosystemic pressure gradient (mmHg); PV‐pressure, portal venous pressure (mmHg); TIPS, Transjugular intrahepatic portosystemic shunt.

**TABLE 2:** 3D TIPS geometry parameters obtained by CT and procedure data.

Parameter		All (*n* = 107)	No dysfunction (*n* = 75)	TIPS dysfunction (*n* = 32)	*p*
3D TIPS geometry	3D Cranial TIPS stent end (mm)	9.99 (2.00–17.93)	6.00 (1.97–14.12)	16.49 (12.24–26.51)	< 0.001
	3D Minimal stent diameter (mm)	8.27 (7.71–9.01)	8.24 (7.66–9.05)	8.32 (7.73–8.87)	0.996
	3D Length of covered stent portion (mm)	63.03 (55.01–72.00)	66.02 (58.03–72.97)	55.45 (47.98–69.28)	0.001
	3D Stent curvature (degrees)	42.12 (36.47–50.37)	40.68 (35.67–49.96)	47.21 (41.30–54.94)	0.017
	3D Angle between the covered stent ends (degrees)	81.60 (66.88–98.73)	81.96 (66.61–102.9)	80.52 (66.89–92.51)	0.414
	3D α Angle (degrees)	53.71 (40.64–64.92)	51.86 (42.36–62.61)	58.48 (39.81–69.5)	0.406
	3D Confluence to TIPS stent (mm)	60.02 (48.02–77.04)	57.94 (46.94–75.08)	66.50 (53.80–80‐44)	0.058
Procedure data	Nominal stent length (cm)	7 (6–8)	8 (7–8)	6 (6–8)	0.004
	Nominal stent diameter (mm)	10 (10–10)	10 (10–10)	10 (10–10)	0.116
	Stent dilatation after TIPS procedure (mm)	8 (8–8)	8 (8–8)	8 (8–8)	0.218
	Underdilated TIPS	90 (86.5%)	66 (89.2%)	24 (80.0%)	0.214
	Type of stent (VTS/VCX)	61/46 (57%/43%)	41/34 (54.7%/45.3%)	20/12 (62.5%/37.5%)	0.454

*Note:* Categorical variables are presented in absolute cases and per cent, while continuous variables are presented as median with interquartile range. Nonparametric testing (Qui‐square test for categorical variables and Mann–Whitney U test for continuous variables). 3D cranial TIPS stent end (mm): Length from the cranial TIPS stent end in the liver vein to the IVC; 3D minimal stent diameter (mm): Smallest diameter in the entire TIPS stent; 3D Length of covered stent portion (mm): Length from the beginning of the distal covered stent end to the end of the cranial covered stent end; 3D stent curvature (degrees/cm): Result of segmentation of the path within the TIPS stent into straight lines with gaps of 5 mm and angle measurements over 1 cm. The parameter describes the maximum change in direction between these sections within the entire stent; 3D angle between the covered stent ends (degrees): Angle between two straight lines orthogonal to the covered stent ends; 3D α angle (degrees): Angle formed between the beginning of the covered stent in the PV and the course of the PV; 3D confluence to TIPS stent (mm): Length from the beginning of the path to the beginning of the uncovered distal stent end.

Abbreviations: TIPS, transjugular intrahepatic portosystemic shunt; VCX, VIATORR controlled expansion stent; VTS: VIATORR TIPS stent.

A total of 73 patients (68,2%) presented with overt HE after the TIPS procedure during the entire follow‐up, while 57 patients (53,3%) presented with overt HE within 12 months after the TIPS procedure. The median time to overt HE in the entire cohort was 49 days. Interestingly, there were no significant differences regarding parameters of 3D TIPS geometry or procedure data between two groups (Table S1).

### 3.2 3D Assessment From CT Reconstruction

The median 3D stent curvature was 42.12 (36.47–50.37) degrees. The median angle between the covered stent ends was 81.60 (68.88–98.73) degrees, and the median length from the cranial TIPS stent end to the IVC was 9.99 (2.00–17.93) mm. The median length from the portal venous confluence to the caudal TIPS stent was 60.02 (48.02–77.04) mm, while the median length of the covered stent portion was 63.03 (55.01–72.00) mm. The median minimum stent diameter was 8.27 (7.71–9.01) mm and the median 3D α angle was 53.71 (40.64–64.92) degrees.

In the TIPS dysfunction group, the distance of the cranial TIPS stent end to the IVC (16.49 vs. 6.00; *p* < 0.001) and the 3D stent curvature (47.21 vs. 40.68, *p* = 0.017) were significantly larger compared to the dysfunction‐free group. Moreover, the length of the covered stent portion was significantly shorter in the TIPS dysfunction group (55.45 vs. 66.02; *p* = 0.001) (Table 2).

### 3.3 Predictors of TIPS Dysfunction

An univariate Cox regression analysis with TIPS dysfunction as the endpoint was performed. The 3D stent curvature (*p* = 0.002, HR 1.019, 95% CI 1.007–1.032) and the 3D cranial TIPS stent end (*p* < 0.001, HR 1.057, 95% CI 1.026–1.090) were significantly associated with TIPS dysfunction in the univariate approach. The 3D length of the covered stent portion (*p* = 0.066, HR 0.967, 95% CI 0.934–1.002) closely failed to reach statistical significance and was also included in the multivariate analysis. Regarding procedure data, the nominal stent length (*p* = 0.062, HR 0.741, 95% CI 0.541–1.016) as well as the status of underdilation of the TIPS (*p* = 0.069, HR 0.429, 95% CI 0.173–1.069) were also included in the multivariate analysis. Of note, data on haemodynamics like central venous (CV) pressure, portal venous (PV) pressure and PSPG before and after TIPS insertion were not associated with the development of TIPS dysfunction.

In the multivariate analysis, only the 3D stent curvature (*p* = 0.003, HR 1.020, 95% CI 1.007–1.034) and the 3D cranial TIPS stent end (*p* < 0.001, HR 1.061, 95% CI 1.030–1.093) were significantly associated with the development of TIPS dysfunction (Table 3). Therefore, a more pronounced curvature as well as a longer distance of the cranial stent end to the VCI were found to be independent predictors of TIPS dysfunction.

**TABLE 3:** Uni‐ and multivariate Cox regression analysis. Endpoint: TIPS dysfunction.

Parameter		*p*	Univariate HR	95% CI	*p*	Multivariate HR	95% CI
3D CT evaluation	3D Cranial TIPS stent end (mm)	< 0.001	1.057	1.026–1.090	< 0.001	1.061	1.030–1.093
	3D Minimal stent diameter (mm)	0.352	0.797	0.495–1.285			
	3D Length of covered stent portion	0.066	0.967	0.934–1.002			
	3D Stent curvature (degrees)	0.002	1.019	1.007–1.032	0.003	1.020	1.007–1.034
	3D Angle between the covered stent ends (degrees)	0.334	0.992	0.977–1.008			
	3D α Angle (degrees)	0.763	1.003	0.984–1.022			
	3D Confluence to TIPS stent (mm)	0.375	1.007	0.991–1.024			
Procedure data	Nominal stent length (cm)	0.062	0.741	0.541–1.016			
	Nominal stent diameter (mm)	0.199	0.517	0.189–1.414			
	Stent dilatation after TIPS procedure (mm)	0.145	1.437	0.882–2.340			
	Underdilated TIPS (yes)	0.069	0.429	0.173–1.069			
Scores	Child–Pugh Score before TIPS	0.627	0.950	0.773–1.168			
	MELD before TIPS	0.917	0.995	0.901–1.098			
Hepatic haemodynamics	CV pressure before TIPS (mmHg)	0.721	0.984	0.902–1.074			
	PV pressure before TIPS (mmHg)	0.888	1.005	0.944–1.069			
	PSPG before TIPS (mmHg)	0.592	1.018	0.955–1.084			
	CV pressure after TIPS (mmHg)	0.850	0.992	0.917–1.074			
	PV pressure after TIPS (mmHg)	0.353	1.035	0.962–1.113			
	PSPG after TIPS (mmHg)	0.119	1.070	0.983–1.165			

*Note:* 3D cranial TIPS stent end (mm) Length from the cranial TIPS stent end in the liver vein to the IVC; 3D Minimal stent diameter (mm): Smallest diameter in the entire TIPS stent; 3D Length of covered stent portion (mm): Length from the beginning of the distal covered stent end to the end of the cranial covered stent end; 3D stent curvature (degrees/cm): Result of segmentation of the path within the TIPS stent into straight lines with gaps of 5 mm and angle measurements over 1 cm. The parameter describes the maximum change in direction between these sections within the entire stent; 3D angle between the covered stent ends (degrees): Angle between two straight lines orthogonal to the covered stent ends; 3D α angle (degrees): Angle formed between the beginning of the covered stent in the PV to the course of the PV; 3D confluence to TIPS stent (mm): Length from the beginning of the path to the beginning of the uncovered distal stent end.

Abbreviations: CI, confidence interval; CV, central venous; HR, hazard ratio; MELD, model for end‐stage liver disease; PSPG, portosystemic pressure gradient; PV, portal venous; TIPS, transjugular intrahepatic portosystemic shunt.

To address the issue of differing time points for CT scans after TIPS insertion used in assessing 3D TIPS geometry, we conducted an additional analysis that considered the time from TIPS insertion to CT acquisition. In the univariate analysis, a significant association was observed between the time from TIPS insertion to CT acquisition and TIPS dysfunction (*p* = 0.027, HR 0.999, 95% CI 0.998–1.000). However, it is worth noting that in the multivariate analysis, the only parameters found to be associated with TIPS dysfunction were once again 3D stent curvature (*p* = 0.004, HR 1.020, 95% CI 1.007–1.034) and 3D cranial TIPS stent end (*p* < 0.001, HR 1.055, 95% CI 1.025–1.087) (Table 4).

**TABLE 4:** Additional uni‐ and multivariate Cox regression analyses including the significant parameters of the multivariate analysis of Table 3 and time from TIPS insertion to CT acquisition. Endpoint: TIPS dysfunction.

Parameter	*p*	Univariate HR	95% CI	*p*	Multivariate HR	95% CI
3D Cranial TIPS stent end (mm)	< 0.001	1.057	1.026–1.090	< 0.001	1.055	1.025–1.087
3D Stent curvature (degrees)	0.002	1.019	1.007–1.032	0.004	1.020	1.007–1.034
Time from TIPS insertion to CT acquisition	0.027	0.999	0.998–1.000			

*Note:* 3D cranial TIPS stent end (mm): Length from the cranial TIPS stent end in the liver vein to the IVC; 3D stent curvature (degrees/cm): Result of segmentation of the path within the TIPS stent into straight lines with gaps of 5 mm and angle measurements over 1 cm. The parameter describes the maximum change in direction between these sections within the entire stent.

Abbreviations: CI, confidence interval; HR, hazard ratio; TIPS, transjugular intrahepatic portosystemic shunt.

### 3.4 Predictors of Overt HE

To investigate risk factors for the development of overt hepatic encephalopathy (HE) following TIPS insertion, we performed an additional Cox regression analysis with overt HE as the endpoint. This analysis included the parameters previously identified as being associated with TIPS dysfunction. In the univariate model, the 3D cranial TIPS stent end was significantly inversely associated with the development of overt HE (*p* = 0.024, HR 0.973, 95% CI 0.950–0.996). In contrast, 3D stent curvature was not significantly associated with the development of overt HE following TIPS insertion in the univariate analysis (*p* = 0.132, HR 0.983, 95% CI 0.961–1.005) (Table S2).

### 3.5 Subgroup Analysis—TIPS for Ascites

Additionally, a subgroup analysis was conducted in patients receiving TIPS for refractory ascites. Detection of ascites more than 30 days after TIPS insertion was the endpoint. Of the 60 patients, 19 (31.7%) ascites could be detected more than 30 days after TIPS insertion at a median time of 48 days. However, although 21 patients (35%) received invasive TIPS revision, ascites could only be detected in 11 patients (18.3%) before TIPS revision. Interestingly, none of the parameters were found to be significantly associated with the detectability of ascites at least 30 days after TIPS insertion (Table S3).

## 4 Discussion

This study demonstrates for the first time that a semiautomated assessment of 3D TIPS geometry can predict TIPS dysfunction and the need for revision. In our study, two parameters were linked to the development of TIPS dysfunction. First, a longer distance of the cranial stent end to the IVC. This result is in line with our and other previous publications on 2D TIPS geometry [18] and relevant for clinical practice because the cranial stent end position can be influenced by the interventionalist's choice of a stent with an adequate length and positioning of the stent. Secondly, and as the key novel finding of our study, we identified a more pronounced curvature as linked to the development of TIPS dysfunction. In this regard, we suggest inserting the TIPS more centrally to avoid a larger curvature of the stent and consecutive potentially problematic haemodynamics of the stent, which might be associated with the development of TIPS dysfunction.

In recent years, several studies evaluated predictors of TIPS dysfunction that are related to TIPS geometry and position. However, the methodology in the current literature is inhomogeneous. Some studies evaluated two‐dimensional imaging such as angiograms, while few others evaluated CT scans. Therefore, the proposed predictors are diverse, including the use of bare metal compared to covered stents, interventionalist's experience [16] and portal venous flow post TIPS [17] as well as α angle or stent position [24, 25]. Except for the use of bare metal stents compared to covered stents, generally, validation for these proposed parameters as predictors of TIPS dysfunction is lacking [10, 12, 14].

Our study depicts the predictive value of 3D TIPS geometry parameters. The analysis of 3D imaging might be more suited to adequately represent the complex anatomy than the analysis of 2D angiography images. A major limitation of the analysis of 2D images is the insufficient representation of the complex portal venous and hepatic anatomy [26]. An optimal 2D angiography angle for TIPS projection is not consented. In a study, the mean optimal projection view for TIPS stent placement was 27° right oblique and 3°craniocaudal. However, even this optimal projection angle showed poor intra‐ and interobserver variability of only 0.6 and 0.48, respectively [27].

Apart from the heterogeneous imaging modalities to evaluate the impact of TIPS geometry on TIPS dysfunction, measuring those parameters (angles, lengths, distance to IVC, etc.) requires time‐consuming manual labour from a trained healthcare specialist. While algorithm‐based automated image analysis is being intensively studied in other areas of liver cirrhosis, such as in the automated assessment of sarcopenia [7, 28, 29] or spontaneous portosystemic shunts [30, 31], there are currently no studies evaluating the computer‐assisted, (semi‐)automated assessment of TIPS geometry. This study demonstrates a modern approach and shows the predictive value of 3D TIPS geometry for the first time. With our algorithm, it is potentially feasible for the clinician to evaluate 3D TIPS geometry in clinical practice in order to stratify patients at risk for TIPS dysfunction. However, we do not suggest routinely performing CT scans after TIPS insertion outside of routine clinical practice to evaluate TIPS geometry because this would mean the need for a second intervention with potential risk and cost factors. A more easily available alternative to facilitate 3D TIPS geometry measurements after TIPS insertion could be to perform routine cone beam CT at the end of the TIPS insertion procedure. However, this study was not intended to encourage routine measurements of 3D TIPS geometry postinsertion but rather to demonstrate the role of 3D geometry regarding the prediction of TIPS dysfunction. Instead of routinely assessing 3D TIPS geometry after insertion, we suggest taking the parameters that we were able to identify as risk factors for the development of TIPS dysfunction into consideration. In terms of the applicability of our data for routine clinical practice and as mentioned earlier, we suggest inserting the TIPS stent into a more central portal venous branch and choosing a stent that is long enough to reach the IVC junction to reduce the risk for TIPS dysfunction. However, one of the main complications that can occur after TIPS implantation is HE. Interestingly, in our separate analyses with overt HE after TIPS insertion as an endpoint, a shorter distance of the cranial TIPS stent end was associated with overt HE after TIPS insertion while the stent curvature was not significantly associated with overt HE after TIPS insertion. Thus, the optimal position of the cranial TIPS stent end regarding risk minimisation for both TIPS dysfunction as well as the development of overt HE after TIPS still has to be determined. Even though this is a comprehensive and so far unique approach and analysis, there are some limitations. The main limitation is the retrospective and monocentric character of the study limiting its generalisability as well as the small number of patients. Additionally, it is important to acknowledge that the stent grafts utilised in this study are a mix of VIATORR controlled expansion stent grafts (VCX) and VIATORR TIPS endoprosthesis stent grafts (VTS). VCX stents were introduced during the study's inclusion period, and the analysis includes both VTS and VCX stents. Notably, the distribution of these two types of stent grafts was similar between the TIPS dysfunction and nondysfunction groups. Therefore, we believe that the findings of our study remain relevant and applicable between the two stent types. Furthermore, we are not able to provide data on heart failure following TIPS implantation because this was not part of the routinely assessed data. The subgroup analysis of patients who received TIPS for refractory ascites revealed no significant association of 3D TIPS geometry with detectable ascites at least 30 days following TIPS insertion. This finding may be attributable to the relatively small sample size within this subgroup. Overall, detectable ascites after TIPS insertion might not necessarily only be related to TIPS dysfunction and could potentially be multifactorial, for example, due to cardiac dysfunction or differences in terms of adherence to diuretic medication.

However, all studies on this topic suffer from a nonstandardised TIPS procedure performed across the world [32]. Mainly, the clinical practice of anticoagulation during and after the TIPS procedure is still debated, and no general consensus exists, despite this factor being potentially crucial for the development of TIPS dysfunction by in‐stent thrombosis. Finally, our algorithm does not account for spontaneous portosystemic shunts (SPSS). SPSS redirect portal venous flow, and the total SPSS area (TSA) have been identified as a significant factor for the outcome of cirrhotic patients [30, 33]. In the context of TIPS, SPSS may function as a competing shunt impacting its function [34]. However, no significant impact on TIPS dysfunction was observed in a recent large cohort [35].

In conclusion, this study shows a modern approach of a computer‐assisted semiautomated assessment of 3D TIPS stent geometry demonstrating its predictive value for the development of TIPS dysfunction. Overall, we were able to identify a larger stent curvature as well as a larger distance of the cranial TIPS stent end to the IVC as risk factors for the development of TIPS dysfunction, both of which can potentially be addressed by clinical measures.

## Author Contributions

**Carsten Meyer:** conceptualization, investigation, data curation, writing – review and editing, methodology, supervision. **Markus Kimmann:** conceptualization, investigation, formal analysis, writing – original draft, methodology, data curation, visualization, validation. **Katharina Böhm:** data curation, conceptualization, writing – original draft, investigation, methodology, formal analysis, validation, visualization. **Sebastian Nowak:** data curation, investigation, writing – review and editing. **Alba Maria Paar Pérez:** investigation, writing – review and editing, data curation. **Jörn Arne Meier:** investigation, writing – review and editing. **Sara Noemi Reinartz Groba:** investigation, writing – review and editing. **Juliana Gödiker:** investigation, writing – review and editing. **Frank Erhard Uschner:** investigation, writing – review and editing. **Feras Sanoubara:** investigation, writing – review and editing. **Johannes Chang:** data curation, investigation, writing – review and editing. **Jonel Trebicka:** conceptualization, project administration, writing – review and editing, investigation. **Alois Martin Sprinkart:** methodology, conceptualization, investigation, supervision, data curation, resources, software, visualization, project administration. **Michael Praktiknjo:** conceptualization, investigation, data curation, supervision, methodology, writing – original draft, resources, funding acquisition, formal analysis, visualization, validation, project administration.

## Ethics Statement

The ethical review board of the University Hospital of Bonn approved the study (positive ethical review board vote 038/20).

## Consent

Written, informed consent was obtained from each patient included in the study.

## Conflicts of Interest

The authors declare no conflicts of interest.

## Authors Statement

All authors approved the final version of the article, including the authorship list.

## Supporting information


Data S1.

Tables S1‐S3.

## Data Availability

The data that support the findings of this study are available from the corresponding author upon reasonable request.
