# Supplementary material for: Three‐Dimensional Transjugular Intrahepatic Portosystemic Shunt Geometry Predicts Shunt Dysfunction
Source: Aliment Pharmacol Ther. 2025 Apr 9;61(11):1805–14. doi: 10.1111/apt.70133 (PMC12074561; doi:10.1111/apt.70133)
Supplement: Supplementary file 1 — Data S1. [file APT-61-1805-s002.docx]

**Supplementary material**

**Supplementary Material 1**

Explanation of the algorithmic approach for 3D TIPS geometry:

Starting from the manually defined path from the portal vein confluence trough the TIPS tract to the inferior vena cava, multi-planar reconstructions (MPR) orthogonal to the path were generated every millimeter along the path. A threshold-based, specially developed algorithm for stent detection was then applied to each individual MPR slice. This algorithm searches radially over 360 degrees around the path and up to a distance of 10 mm for voxels with a density of 250 Hounsfield units or higher. If at least one such voxel is found in each quadrant, the corresponding path position is labelled as being within the stent. The venous end of the stent is automatically identified by the high-density clip. The transition between the covered and the uncovered part of the stent is detected by the high-density ring marking.

To ensure high accuracy of the subsequent analyses of various angles as described below, a circular function is fitted to the detected stent voxels and the manually defined path is then shifted to the center of the fitted circular function. The circular fit function is also used for determination of the minimal stent diameter.

To calculate the maximum kink angle in the stent (3D Stent Curvature), 2 areas along the path were linearized over 5 mm of the path at 1 cm distance from each other. This process was performed over the entire length of the stent at 1 mm intervals to calculate the maximum kink of the stent in degrees. To determine the angle between the portal venous stent end and the portal vein (3D α angle), the stent end was linearized over the last 5 mm and the path in the portal vein was linearized over a range of 2 cm at a distance of 3 cm from the stent end. For all other angle measurements, the path was linearized over 5 mm at the respective positions.
The evaluation was carried out using dedicated evaluation software written in MATLAB (Mathworks).
