# Supplementary material for: Three‐Dimensional Transjugular Intrahepatic Portosystemic Shunt Geometry Predicts Shunt Dysfunction
Source: Aliment Pharmacol Ther. 2025 Apr 9;61(11):1805–14. doi: 10.1111/apt.70133 (PMC12074561; doi:10.1111/apt.70133)
Supplement: Supplementary file 2 — Tables S1‐S3. [file APT-61-1805-s001.zip › Supplemental Tables.docx]

| **Parameter** | | **all (n = 107)** | **HE after TIPS** (n = 73) | **No HE after TIPS** (n = 34) | **P** |
| --- | --- | --- | --- | --- | --- |
| **3D TIPS Geometry** | 3D Cranial TIPS stent end (mm) | 9.99 (2.00-17.93) | 9.94 (2.00-15.89) | 10.48 (1.74-25.50) | 0.315 |
|  | 3D Length of covered stent portion (mm) | 63.03 (55.01-72.00) | 62.01 (54.99-71.52) | 68.50 (55.74-72.96) | 0.181 |
|  | 3D Stent Curvature (degrees) | 42.12 (36.47-50.37) | 42.53 (36.18-50.08) | 42.05 (37.58-54.97) | 0.606 |
|  | 3D Angle between the covered stent ends (degrees) | 81.60 (66.88-98.73) | 88.52 (68.71-99.52) | 76.66 (62.34-98.95) | 0.297 |
|  | 3D α Angle (degrees) | 53.71 (40.64-64.92) | 54.38 (39.19-64.75) | 51.87 (43.04-70.77) | 0.723 |
|  | 3D Confluence to TIPS stent (mm) | 60.02 (48.02-77.04) | 63.81 (49.05-78.49) | 57.48 (47.70-77.01) | 0.366 |
| **Procedure Data** | Nominal stent diameter (mm) | 10 (10-10) | 10 (10-10) | 10 (10-10) | 0.495 |
|  | Stent Dilatation after TIPS procedure (mm) | 8 (8-8) | 8 (8-8) | 8 (8-8) | 0.235 |
|  | Underdilated TIPS (yes/no) | 90/14 (86.5%/13.5%) | 63/8 (88.7%/11.3%) | 27/6 (81.8%/18.2%) | 0.336 |

**Supplemental Table S1. Study population characteristics at baseline (divided be development of overt HE after TIPS).** TIPS: Transjugular intrahepatic portosystemic shunt. Categorical variables are presented in absolute cases and percent while continuous variables are presented as median with interquartile range. Non-parametric testing (Qui Square Test for categorical variables, Mann Whitney U Test for continuous variables).

| **Parameter** | |  | | | | | |
| --- | --- | --- | --- | --- | --- | --- | --- |
|  |  | **P** | **Univariate  HR** | **95 % CI** | **P** | **Mutivariate HR** | **95 % CI** |
| **3D CT evaluation** | 3D cranial TIPS stent end (mm) | 0.024 | 0.973 | 0.950-0.996 | 0.024 | 0.973 | 0.950-0.996 |
|  | 3D Stent Curvature (degrees) | 0.132 | 0.983 | 0.961-1.005 |  |  |  |

**Supplemental Table S2. Additional uni- and multivariate Cox regression analysis with overt HE after TIPS including the significant parameters of the multivariate analysis of Table 3.** Endpoint: Overt HE after TIPS. HR: Hazard ratio; CI: Confidence interval; TIPS: Transjugular intrahepatic portosystemic shunt; 3D cranial TIPS stent end (mm): length from the cranial TIPS stent end in the liver vein to the IVC; 3D Stent Curvature (degrees/cm): Result of segmentation of the path within the TIPS stent into straight lines with gaps of 5 mm and angle measurements over 1 cm. The parameter describes the maximum change in direction between these sections within the entire stent.

| **Parameter** | |  | | | | | |
| --- | --- | --- | --- | --- | --- | --- | --- |
|  |  | **P** | **Univariate  HR** | **95 % CI** | **P** | **Mutivariate HR** | **95 % CI** |
| **3D CT evaluation** | 3D cranial TIPS stent end (mm) | 0.575 | 1.013 | 0.867-1.062 |  |  |  |
|  | 3D Minimal stent diameter (mm) | 0.917 | 0.971 | 0.564-1.674 |  |  |  |
|  | 3D Length of covered stent portion | 0.840 | 0.996 | 0.956-1.038 |  |  |  |
|  | 3D Stent Curvature (degrees) | 0.918 | 1.002 | 0.961-1.045 |  |  |  |
|  | 3D Angle between the covered stent ends (degrees) | 0.988 | 1.000 | 0.980-1.020 |  |  |  |
|  | 3D α Angle (degrees) | 0.771 | 1.004 | 0.978-1.031 |  |  |  |
|  | 3D Confluence to TIPS stent (mm) | 0.586 | 1.005 | 0.986-1.025 |  |  |  |
| **Procedure data** |  |  |  |  |  |  |  |
|  | Nominal stent length (cm) | 0.449 | 0.859 | 0.579-1.274 |  |  |  |
|  | Nominal stent diameter (mm) |  |  |  |  |  |  |
|  | Stent Dilatation after TIPS procedure (mm) | 0.915 | 1.034 | 0.556-1.923 |  |  |  |
|  | Underdilated TIPS (yes) | 0.915 | 0.935 | 0.270-3.233 |  |  |  |
| **Scores** | Child Pugh Score before TIPS | 0.080 | 0.733 | 0.517-1.038 |  |  |  |
|  | MELD before TIPS | 0.522 | 1.040 | 0.923-1.171 |  |  |  |
|  |  | | | |  | | |
| **Hepatic hemodynamics** |  | | | | | | |
|  | CV pressure before TIPS (mmHg) | 0.709 | 0.980 | 0.884-1.087 |  |  |  |
|  | PV pressure before TIPS (mmHg) | 0.391 | 0.970 | 0.905-1.040 |  |  |  |
|  | PSPG before TIPS (mmHg) | 0.556 | 0.978 | 0.910-1.052 |  |  |  |
|  | CV pressure after TIPS (mmHg) | 0.886 | 1.008 | 0.914-1.112 |  |  |  |
|  | PV pressure after TIPS (mmHg) | 0.628 | 0.979 | 0.897-1.068 |  |  |  |
|  | PSPG after TIPS (mmHg) | 0.379 | 0.944 | 0.830-1.074 |  |  |  |

**Supplemental Table S3. Cox regression analysis including patients receiving TIPS for refractory ascites. Endpoint: Recurrence of ascites after TIPS.** HR: Hazard ratio; CI: Confidence interval; TIPS: Transjugular intrahepatic portosystemic shunt; MELD: Model for End-stage Liver Disease; CV: central venous; PV: portal venous; PSPG: portosystemic pressure gradient; 3D cranial TIPS stent end (mm): length from the cranial TIPS stent end in the liver vein to the IVC; 3D Minimal stent diameter (mm): smallest diameter in the entire TIPS stent; 3D Length of covered stent portion (mm): length from the beginning of the distal covered stent end to the end of the cranial covered stent end; 3D Stent Curvature (degrees/cm): Result of segmentation of the path within the TIPS stent into straight lines with gaps of 5 mm and angle measurements over 1 cm. The parameter describes the maximum change in direction between these sections within the entire stent; 3D Angle between the covered stent ends (degrees): angle between two straight lines orthogonal to the covered stent ends; 3D α Angle (degrees): angle formed between beginning of the covered stent in the PV to the course of the PV; 3D Confluence to TIPS stent (mm): length from the beginning of the path to the beginning of the uncovered distal stent end.
